# Nutritrauma: A Key Concept for Minimising the Harmful Effects of the Administration of Medical Nutrition Therapy

**DOI:** 10.3390/nu11081775

**Published:** 2019-08-01

**Authors:** Juan Carlos Yébenes, Lluis Campins, Itziar Martínez de Lagran, Lluisa Bordeje, Carol Lorencio, Teodoro Grau, Juan Carlos Montejo, Maria Bodí, Mateu Serra-Prat, Working Group on Nutrition and Metabolism of the Spanish Society of Critical Care

**Affiliations:** 1 Servei de Medicina Intensiva, Hospital de Mataró, 08304 Mataró, Spain; 2 Grup de Recerca en Sèpsia, Inflamació i Seguretat del Pacient Crític, 43005 Tarragona, Spain; 3 Servei de Farmacia, Hospital de Mataró, 08304 Mataró, Spain; 4 Servei de Medicina Intensiva, Hospital Universitari Germans Trias i Pujol, 08916 Badalona, Spain; 5 Servei de Medicina Intensiva, Hospital Universitari de Girona Dr. Josep Trueta, 17007 Girona, Spain; 6 Servicio de Medicina Intensiva, Hospital Universitario 12 de Octubre, 28041 Madrid, Spain; 7 Servei de Medicina Intensiva, Hospital Universitari de Tarragona Joan XXIII, 43005 Tarragona, Spain; 8 Unitat de Recerca, Hospital de Mataró, 08304 Mataró, Spain

**Keywords:** medical nutrition therapy, enteral nutrition, parenteral nutrition, nutritional support, critical care, iatrogenesis

## Abstract

Critically ill patients often require life support measures such as mechanical ventilation or haemodialysis. Despite the essential role of nutrition in patients’ recovery, the inappropriate use of medical nutrition therapy can have deleterious effects, as is the case with the use of respiratory, circulatory, or renal support. To increase awareness and to monitor the effects of inappropriate medical nutrition therapy, we propose to introduce the concept of nutritrauma in clinical practice, defined as metabolic adverse events related to the inappropriate administration of medical nutrition therapy or inadequate nutritional monitoring.

## 1. Introduction

During the twentieth century, many medical interventions were introduced to treat clinical conditions that would previously have been fatal. The introduction of mechanical ventilation and haemodialysis increased the chance of survival among people with respiratory failure and renal failure. Far from being physiological, these life-support techniques can have adverse effects, which can produce activation of the inflammatory cascade, microscopic lesions at the cellular level, or precipitate organ failure [1,2].

The study of the clinical effects and outcomes following the administration of life support techniques has led to the development of safer and better-tolerated therapies [3,4]. In the case of mechanical ventilation, replacing the cycles of positive and negative pressure of spontaneous ventilation with positive pressure throughout the breath cycle produces a decrease in venous return, alterations in the immune response, and traumatic injuries due to hyperpressure (barotrauma), volutrauma, and atelectrauma (Table 1). Mechanical ventilation has provided life support for many patients who are in critical condition, but experience has taught us that it is insufficient to only program a suitable respiratory rate, tidal volume, and fraction of inspired oxygen (FiO_2_), but that it also requires choosing when to intubate, providing adequate sedation, the use of volume and pressure alarms, making postural changes, aspiration of secretions, and measures to prevent infections induced by the ventilation. The value of mechanical ventilation in intensive care units is not open to question. Without questioning its usefulness, we can continue to try to improve the ventilatory support for patients who cannot breathe, while minimising the adverse effects of its use [5].

## 2. Medical Nutrition Therapy as a Form of Life Support

**Figure 1:**
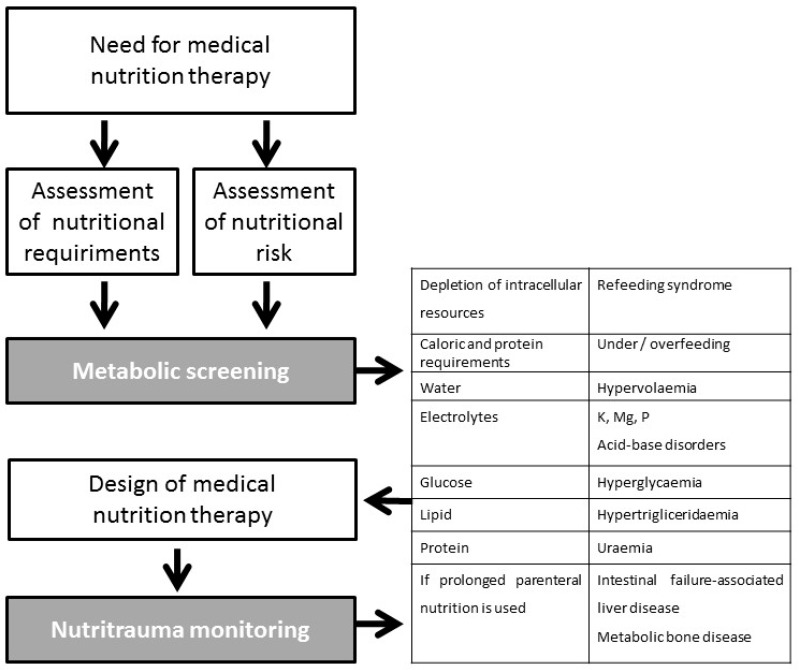
Schema for incorporating the concept of nutritrauma into clinical practice.

People need nutrients to stay alive. Maintaining the structure and function of the human body depends on the intake of energy and nutrient components. Therefore, in patients who cannot take nutrients due to anatomical or functional insufficiency, medical nutrition therapy (MNT) must be provided as a form of life support. However, as with other life support measures, in the design of MNT, the quantity and composition of nutrient intake is determined by the physician and not by the patient. Moreover, substrate utilisation in critically ill patients is affected by the inflammatory response associated with acute injury [6]. An inadequate assessment of when to initiate nutritional support, the patient’s capacity to manage the support, the route of administration, and the amount or composition or nutritional support, can have harmful consequences for the patient [7]. The iatrogenic effect of inappropriate nutritional support should be monitored [8] and included in health quality standards [9,10]. Use of the umbrella term “nutritrauma” to encompass the broad clinical spectrum of harms that can be caused by nutritional support could facilitate the spread of this concept, increase awareness of the iatrogenic effects of inappropriate nutritional support, and lead to training measures being introduced to minimise its occurrence (Figure 1).

## 3. Complications of Medical Nutrition Therapy that should be Included in the Definition of Nutritrauma

There are many complications of medical nutrition therapy that should be included in the definition of nutritrauma (Table 2). These include the following:

Miscalculation of calorie and protein requirements

Fluid overload

Metabolic complications of electrolyte and acid-base imbalance

Hypertriglyceridemia

Hepatobiliary disorders associated with parenteral nutrition (PN)

Metabolic bone disease

Hyperglycaemia

Food hypersensitivities

Refeeding syndrome

Each of these is discussed below.

### 3.1. Miscalculation of Calorie and Protein Requirements

Under-prescription and over-prescription of nutrients can cause metabolic complications and affect prognosis [11,12]. Overnutrition should be regarded as associated with not only PN but also the metabolic impact of providing direct intravenous infusion of nutrients. Stressed patients have a limited capacity to process water, energy and protein waste. Moreover, insulin resistance is related to inflammatory stress and leads to increased protein metabolism as the primary intracellular energy substrate in place of lipids or glucose. Ketosis is not frequent in patients with a negative protein balance [13]. Metabolic imbalances can be prevented by phasing the introduction of enteral nutrients, while the sudden introduction of parenteral nutrition without adjusting it to the patient’s nutritional requirements can result in metabolic disturbances [14]. Calories and protein should be evaluated and prescribed separately to avoid over- or under-feeding.

In the stressed critically ill patient, the amount of carbohydrate catabolized for metabolic fuel, and CO_2_ production is proportional to the amount administered [15]. CO_2_ production is also related to over-prescription of calories [16] and can produce hypercapnia in patients where the pulmonary reserve is limited, increasing days of mechanical ventilation [17]. Moreover, administration of >100% of the calories of resting energy expenditure, estimated by indirect calorimetry, has been shown to be associated with an increased mortality rate. In patients who received >70% of calories of resting energy expenditure, the number of days of mechanical ventilation that they required and the duration of their stay in the intensive care unit (ICU) were increased [12]. The Tight Calorie Control Study (TICACOS) showed similar results: Patients who received more calories did not have a higher survival rate, but they required more days of mechanical ventilation and stayed longer in the ICU [18]. Moreover, administering calories in excess of a patient’s energy requirements may lead to liver dysfunction and non-alcoholic fatty liver disease [19,20].

Multiple studies and meta-analyses have focused on undernutrition in recent years. These studies were heterogeneous, but none of them showed an increase in survival. In some of the studies in which a protein intake >1 g/kg/d was maintained, the incidence of nosocomial infections decreased, but this had no impact on mortality. Therefore, patients in critical condition require an adequate calorie intake (with indirect calorimetry is used as the gold standard for estimating energy expenditure), and a high protein intake (from 1.2 up to 2 g/kg/d, according to the latest guidelines) [21,22].

### 3.2. Fluid Overload

Fluid overload can be caused by failing to reduce the volume of fluids administered after the patient becomes stabilised. The volume of fluid required must be re-evaluated after prescribing nutritional support. The fluid requirement of adults with a normal hydration status is approximately 30 to 40 ml/kg/d [23], but it also depends on the patient’s underlying clinical condition and needs to be individualised. When calculating the total volume of PN, secondary sources of fluid inputs must be considered. Otherwise, PN can lead to fluid overload (hypervolaemia). A positive fluid balance is known to be associated with higher mortality and morbidity among ICU patients [24]. Patients with pre-existing fluid retention due to underlying cardiac and hepatic disease are more likely to deteriorate in condition while receiving PN [25]. On the other hand, during illness, there are multiple sources of fluid losses, such as nasogastric suctioning, vomiting, diarrhea and fistula drainage. Such losses must be replaced to prevent fluid deficits, and the PN solution must be adjusted accordingly. All patients receiving PN should have their fluid input and output monitored and recorded accurately.

### 3.3. Metabolic Complications of Electrolyte and Acid-Base Imbalance

Potassium is the most abundant intracellular cation. Potassium requirements range from 0.5 to 1.5 mmol/kg/d. Hypokalaemia is the most common electrolyte abnormality and is caused by the displacement of potassium from the extracellular space into the intracellular space as a result of metabolic alkalosis, refeeding syndrome, or the administration of insulin, catecholamines, and other drugs, such as diuretics and glucocorticoids [26]. Hypokalaemia can be treated by increasing intravenous potassium administration. Hypokalaemia is usually associated with hypomagnesaemia, and magnesium should be replenished before administering potassium because magnesium deficiency increases renal potassium losses and disrupts the equilibrium of the sodium–potassium ATPase pump [27]. Conversely, hyperkalaemia is less frequent and is usually related to impaired renal function, but it can also occur as a result of metabolic respiratory acidosis or administration of drugs, such as angiotensin-converting enzyme inhibitors, β-blockers, potassium-sparing diuretics, and nonsteroidal anti-inflammatory drugs [28].

Hypocalcemia is a common finding in critically ill patients. The aetiology of hypocalcemia is uncertain and potentially multifactorial, and the prevalence varies widely depending on the different underlying diseases and comorbidity [29,30]

Magnesium is an important cofactor in more than 3,000 enzyme reactions involving adenosine triphosphate (ATP) [31]. It also regulates the movement of calcium into smooth muscle cells to maintain cardiac contractile strength and peripheral vascular tone. Hypomagnesaemia is commonly observed in critically ill patients, and clinical manifestations include cardiac arrhythmias, muscle weakness, hyperreflexia, and hypokalaemia [26]. Magnesium intake for most patients ranges from 24 to 32 meq/day, whereas higher amounts can be required for patients with diarrhea [32]. Hypermagnesaemia is uncommon and typically only seen in patients with renal insufficiency and in patients who have received high doses of magnesium supplements.

Phosphorus is a major component in ATP production. Providing excessive intravenous dextrose calories, or a large increase in the amount of PN administered leads to increased insulin secretion and redistribution of phosphate from the extracellular fluid to the intracellular space [26]. Common signs of hypophosphataemia include ataxia, confusion, weakness, fatigue, muscle necrosis, and cardiac or respiratory failure. Most critically ill patients require about 0.32 mmol/d. An excess in phosphorus provision besides an impaired excretion due to renal insufficiency can lead to hyperphosphataemia. [25]

Excessive parenteral chloride administration can contribute to metabolic acidosis, while an excess of acetate salts can cause metabolic alkalosis. Although PN should not be used for correcting the acid–base balance, an increase in the intake of chlorine salts helps to correct metabolic alkalosis. Conversely, acetate is converted to the equivalent amount of bicarbonate, so higher levels of acetate salts can correct the bicarbonate deficit in patients with diarrhea and fluid loss through fistulae [33].

### 3.4. Hypertriglyceridaemia

Hypertriglyceridaemia is a well-known metabolic complication of medical nutrition therapy and is primarily associated with fat administration as part of PN. The prevalence of hypertriglyceridaemia ranges from 6% to 38%, depending upon the patient population studied and the definition of hypertriglyceridaemia used [34]. 

Hypertriglyceridaemia is caused not only by an excessive dose of lipids in PN but also by a drug-induced suppression of lipoprotein lipase or stimulation of lipogenesis induced by excessive carbohydrate ingestion. Therefore, hypertriglyceridaemia in patients in critical condition must be understood as an imbalance between fat administration and plasma fat clearance capacity. Several other factors are known to increase the risk of hypertriglyceridaemia during PN. These include the fat-overload syndrome (>3–4 g/Kg of lipids infused), renal failure, administration of high doses of corticosteroids (>0.5 mg/kg/d), sepsis, pancreatitis, hyperglycaemia (>180 mg/dL), whereas heparin infusion (>3 mg/kg/d) has shown protective effects that can be related to lipoprotein lipase stimulation [35]. In critically ill patients, blood triglyceride levels can be reduced by using the latest generation lipids [36]. Propofol is an anaesthetic substance commonly used in critically ill patients and is formulated as a lipid emulsion in 10% soybean oil. The use of propofol can lead to hypertriglyceridaemia, especially in patients with sepsis [37] Devlin et al. [38], reported on a case-series of 159 patients treated with propofol, of whom 18% had hypertriglyceridaemia, and 21% had triglyceride levels >1000 mg/dL.

There is no consensus regarding the classification of levels of hypertriglyceridaemia or methods to manage hypertriglyceridaemia in patients receiving PN. Currently, the recommended lipid intake for patients receiving PN varies from 0.7 to 1.5 g/kg/d in critically ill patients [39]. It has been proposed that the lipid level in PN be reduced if patients have low or moderate plasma triglyceride levels [40] and that lipids be withdrawn in patients with plasma triglyceride levels in the 400 mg/dL [41] to 1000 mg/dL range [42]. When cyclic nutrition is applied, the optimal time to measure triglycerides is the 6 to 8 hours trough, and not while lipids are being infused.

Intestinal failure-associated liver disease (IFALD) is a hepatic dysfunction secondary to intestinal failure in the presence of PN, and it has a range of manifestations. The prevalence of IFALD in adults receiving PN ranges from 15% to 85%, and its occurrence and severity increase with longer duration of PN [43,44].

### 3.5. Hepatobiliary Disorders Associated with Parenteral Nutrition

PN-associated cholestasis (PNAC) is a serious complication which may progress to cirrhosis and to liver failure. Patients with PNAC have an elevated conjugated bilirubin (>2 mg/dL) and increased serum gamma-glutamyltransferase and alkaline phosphatase. Gallbladder stasis is generally related to a lack of enteral stimulation rather than the PN infusion [45]. The aetiology of the hepatic complications in patients receiving PN is unclear, but there are some risk factors that contribute to IFALD. Providing excess nutrients, particularly carbohydrates and lipids results in fat deposition in the liver and contributes to essential fatty acid deficiency, leading to the development of steatosis [46]. Continuous infusion of nutrients can also increase the risk of hepatic complications by causing hyperinsulinaemia [26]. Dosing, source and the phytosterol content of the fat emulsions should all be considered to prevent the development of steatosis and cholestasis [26]. The new generation of fat emulsions containing a combination of soya bean oil, olive oil, fish oil, and medium-chain triglycerides have anti-inflammatory properties, higher antioxidant content, and are less likely to cause cholestasis [46]. In patients receiving PN, other risk factors for liver injury include essential fatty acid deficiency, taurine deficiency and hypermagnesaemia [47].

Prevention of IFALD may include different strategies, such as decreasing the macronutrient content of PN, especially the dextrose content, providing cyclic PN and, most importantly, switching to enteral nutrition as soon as possible [26].

### 3.6. Metabolic Bone Disease

PN is associated with loss of bone calcium and low bone density, with reported prevalence as high as 84% in patients receiving PN for an extended period [48]. The aetiology of metabolic bone disease (MBD) is poorly understood and may be related to various components of PN and patients’ underlying risk factors.

An excess of amino acid in PN, administered during repletion, can cause hypercalciuria and negative total calcium balance. Low calcium and phosphate intake can also lead to the development of MBD. There is a limitation on PN calcium dosing due to physical compatibility of calcium and phosphorus. PN formulations should contain ≥15 mEq/d of calcium and 20 to 40 mmol/d of phosphate to prevent excessive calcium excretion [47,49] 

### 3.7. Hyperglycaemia

Hyperglycaemia is the most common metabolic complication of artificial nutrition. PN formulations are usually initiated at 2 g/kg/d of glucose to prevent the gluconeogenesis derived from amino acid precursors provided by skeletal muscle proteolysis. Administration of >4 mg/kg/min often leads to hyperglycaemia [47]. The prevalence of hyperglycaemia in patients receiving PN is high and has exceeded 40% in some studies [50,51]. In patients receiving PN, hyperglycaemia is associated with increased mortality, infections, organ dysfunction, and longer hospitalisation [52,53].

During the first 24 hours after starting PN, blood glucose should be measured at least 4-hourly in critically ill patients, and the frequency of blood glucose monitoring should only be decreased once the patient has stabilised, usually after about 48 hours [26]. There is a lack of consensus regarding the optimal blood glucose range, especially as tight control is associated with an increased risk of hypoglycaemia and an increased risk of death [54]. The American Society for Parenteral and Enteral Nutrition (ASPEN) recommends that blood glucose be maintained in the 140–180 mg/dL range [22] for adult patients who are critically ill, but the European Society for Clinical Nutrition and Metabolism (ESPEN) does not give any specific recommendation [21].

### 3.8. Hypersensitivity

Hypersensitivity to the components of PN is a rare but an important complication which can cause problems ranging from minor symptoms, such as pruritus, [55] to life-threatening conditions, such as anaphylaxis [56]. In a recent systematic review, the components of PN most frequently identified as allergens were fat emulsions multivitamin solutions [57].

### 3.9. Refeeding Syndrome

Refeeding syndrome is a complex metabolic phenomenon related to the depletion of intracellular resources, minerals (phosphate, potassium, and magnesium), vitamins (thiamine), and trace elements caused by the reactivation of metabolism in previously underfed patients [58]. Patients with a low BMI, unintentional weight loss in the previous few months, little nutritional intake for >10 days, or with abnormal plasma potassium, phosphate, or magnesium levels are at risk of refeeding syndrome after receiving nutritional support. Clinical symptoms of refeeding syndrome vary widely. Phosphate is present in energy transfer reactions, nervous and cardiac conduction systems, and other membrane-mediated cellular functions in the renal, haematological, muscular, and immunological systems. During the transition from PN to regular feeding, intracellular phosphate depletion can be prevented by gradually increasing calorie intake following calorie restriction, thiamine and electrolyte supplementation, and electrolyte monitoring [59].

## 4. Monitoring Requirements

Appropriate design of MNT requires an evaluation of nutritional risk, nutritional access, and quantitative and qualitative composition of the supplemental nutrition components, including water, electrolytes, calories (lipid and non-lipid calories) and protein [6,21,60,61].

To increase awareness regarding inappropriate MNT and to improve monitoring, we propose introducing the word “nutritrauma” as a new term in clinical nutrition [9], defined as metabolic adverse events related to an inadequate nutritional assessment and inappropriate prescription of MNT (in terms of timing, route of administration, and quantitative and qualitative composition). We recommend that non-metabolic complications of supplemental nutrition, such as infections (e.g., catheter-related bacteraemia and nasogastric tube-associated sinusitis), mechanical and vascular complications (e.g., non-occlusive intestinal ischemia), not be included in the definition because these complications require other forms of management and different resources.

Defining nutritrauma monitoring as a key safety indicator would enable nutritional support to be included in quality and safety improvement programmes. Independently of nutritrauma incidence, adding nutritrauma monitoring to such programmes may help to draw attention to the relevance of nutritional support and the need to develop a standardised approach to providing nutritional support, reducing variability and non-evidence-based management. This approach could also enable more professionals to become involved with nutritional management as a life support technique. In Table 3, we propose a definition for “monitoring nutritrauma” as a safety indicator. 

## 5. Conclusions

Is well established that inappropriate MNT is associated with metabolic complications that affect the prognosis of critically ill patients. Introducing the concept of nutritrauma, defined as iatrogenic metabolic effects of MNT, in clinical practice could be a useful means of increasing awareness of the need for thorough nutritional assessment and appropriate prescription of MNT, and could facilitate monitoring strategies, thus reducing adverse events related to nutritional support. Moreover, monitoring the occurrence of nutritrauma should be considered to be a safety indicator. Monitoring and reporting of nutritrauma in critically ill patients should be implemented without delay. 

## Figures and Tables

**Table 1:** Adverse events (AE) and iatrogenic injuries associated with life support techniques.

Item	INVASIVE MECHANICAL VENTILATION	CONTINUOUS HAEMODIALYSIS	MEDICAL NUTRITION THERAPY
Indication	Alteration of ventilation Alteration of oxygenation Airway permeability	Uraemia Dyselectrolytaemia Hypervolemia Removing toxins (endogenous and exogenous) Hypothermia	Inadequate nutritional intake
Access	Tracheal tube Tracheostomy	Central venous line	Central venous line Enteral tube
Adjuvant treatments	Sedation Neuromuscular block Artificial nutrition Physiotherapy	Systemic anticoagulation Circuit heater or thermal blanket	Insulin administration for glycaemic control Physiotherapy
AE related to access	Stenosis, Granulomas, Mucosal necrosis	Hemorrhage Thrombosis	Hemorrhage Thrombosis
AE related to infections	Ventilator-associated pneumonia	Catheter-related infections	Sinusitis Catheter-related infections
AE related to toxicity	Oxygen toxicity	Heparin-induced thrombocytopenia. Citrate-induced hypocalcemia	Chemical phlebitis
AE related to the technique	Barotrauma: Lung injury due to positive pressure, overdistention and alveolar break Volutrauma: Lung injury due to local overdistention as a result of using excessive tidal volumes Atelectrauma: Alveolar damage as a result of transient and repeated closure and reopening of alveoli during the respiratory cycle. Biotrauma: Mechanical stress in response to using high tidal volume and inadequate positive end-expiratory pressure (PEEP) leading to a systemic inflammatory response	Dialytrauma: Harmful effects of continuous renal replacement therapy Dyselectrolytaemia: hypokalaemia, hypophosphataemia, hyponatraemia, hypo/hypercalcaemia, alteration of lactate metabolism Loss of endogenous vital nutrients: water-soluble vitamins, antioxidants, carbohydrates, amino acids Incorrect adjustment of drugs Hypothermia Blood loss: secondary to circuit lifespan	Nutritrauma: Over- and underfeeding, Hypervolaemia Uraemia Hypercapnia Hypertriglyceridaemia Refeeding syndrome Dyselectrolytaemia Hyperglycaemia Hepatobiliary disorders: Cholestasis, cholecystitis, cholelithiasis, hepatic steatosis, non-alcoholic fatty liver disease, hepatic fibrosis and cirrhosis Metabolic bone disease Malabsorptive diarrhea

**Table 2:** Complications of medical nutrition therapy included in the definition of nutritrauma.

Component	Adverse Events	Therapeutic Approach
Inappropriate dosage	Overfeeding, underfeeding	Adjust calorie and protein administration to inflammatory status and clinical phase, Adjust non-nutritional calorie administration, Minimise interruptions and optimise enteral administration
Refeeding syndrome	Depletion of intracellular resources in previously malnourished or fasting patients	Assess nutritional risk and requirements, Restrict calorie intake during the first few days of transitioning back to a normal diet
Water	Fluid overload	Adjust prescription to liquid balances
Electrolytes	Electrolyte disturbances (K, Mg, P, acid-base disorders)	Periodic laboratory tests
Glucose	Hypoglycaemia, hyperglycaemia	Glycaemic control protocol
Calorie intake Lipid	Hypertriglyceridaemia, non-alcoholic fatty liver disease	Adjust lipid administration to calorie requirements, periodic laboratory test
Protein	Uraemia	Adjust protein prescription to requirements and stress
Prolonged use of Parenteral Nutrition	Intestinal failure-associated liver disease, metabolic bone disease	Test enteral tolerance if possible

**Table 3:** Establishing nutritrauma monitoring as a key safety indicator.

Indicator Name	Nutritrauma Monitoring
Area	Safety
Justification	Inappropriate dosage of medical nutrition therapy is associated with greater morbidity and mortality in the critically ill patient. Monitoring the components of nutritional support can assist in recovery and improve prognosis
Formula	(No. of patients monitored) × 100 ÷ (No. of patients with medical nutritional support)
Explanation of terms	Monitoring of nutritrauma includes: An initial assessment of the nutritional status Assessment of calorie and protein requirements Monitoring of the administration of supplemental nutrition Monitoring water balance Monitoring of hypoglycaemia and hyperglycaemia Monitoring the incidence of uraemia not attributable to renal failure Monitoring the incidence of hypolipidaemia and hyperlipidaemia Monitoring the incidence of dyselectrolytaemia Monitoring the incidence of hepatopathy
Population	All critically ill patients who receive medical nutritional support
Type	Process
Data source	Medical records and clinical information systems
Standard	100%
